# Temperature-dependent synthesis of dimethyl ether (DME) from methanol over beta zeolite: a novel approach to a sustainable fuel

**DOI:** 10.1098/rsos.230524

**Published:** 2023-08-23

**Authors:** Puneet Kumar Chaudhary, Racha Arundhathi, Mahesh W. Kasture, Chanchal Samanta, Rakesh Vankayala, Chiranjeevi Thota

**Affiliations:** Corporate Research & Development Centre, Bharat Petroleum Corporation Limited, Greater Noida, Uttar Pradesh 201306, India

**Keywords:** methanol dehydration, DME, olefins, zeolite, porosity

## Abstract

Crystalline beta zeolite molecular sieve with SiO_2_/Al_2_O_3_ molar ratio of 28.5 was synthesized by the hydrothermal crystallization method and examined for methanol dehydration reaction. The micro-mesoporous beta zeolite was active between 280 and 450°C. Dimethyl ether (DME) was observed as the predominant product at all reaction temperatures, with a maximum selectivity of 47.9% at 300°C and a methanol turnover frequency (TOF_MeOH_) of 741.9 h^−1^. At increased reaction temperatures, beta zeolite showed enhanced strong acid site fraction, promoting higher hydrocarbon formation following the olefin-based cycle. It was revealed that the crystallinity, porosity and acidity of beta zeolite change in the reaction environment. Amorphous carbon deposition occurred on beta zeolite, which involved a loss in crystallinity to some extent. The temperature increase showed a pore-broadening phenomenon at elevated temperature regions. Regeneration cycle testing demonstrated beta zeolite activity maintained stable throughout a 280 h time-on-stream period.

## 1. Introduction

Global population growth, rapid urbanization and the use of fossil fuels like coal, oil and natural gas for economic purposes have all contributed to excessive CO_2_ emissions and subsequent climate change. As a result, there is an urgent need to promote the use of renewable energy sources that can replace fuels and chemicals made from fossil fuels in order to prevent the harmful effects of climate change. Also, the circular economy has become crucial for sustainable development to reduce carbon footprint by the use of abundant renewable resources like solar, wind and biobased resources.

Recently, countries around the world have put significant focus on developing technologies for the production of synthetic fuels from renewable sources to reduce carbon emissions from the transportation sector. As India is an high energy demanding country and heavily dependent on imported fossil fuels, the government is promoting synthetic fuels and biofuels to become more self-reliant. India is also a signatory to the Paris Agreement and is committed to reducing its carbon footprint from its economic activities. During COP26, the Indian government committed to becoming a carbon-neutral country by 2070 [1].

In the pursuit of carbon neutrality, dimethyl ether (DME) has emerged as a chemically stable and environmentally viable compound. Given its comparable properties to LPG and diesel fuels, DME is garnering significant attention as a potential fuel additive [2,3]. Currently, DME synthesis is preferably carried out by methanol dehydration (Reaction 1.1). The exothermic nature of the reaction leads to the formation of hot spots, which pose a significant risk of catalyst damage [4]. The presence of carbon deposition poses an inherent challenge by impeding the accessibility of acidic sites [5]. Additionally, the water produced during the process exhibits a strong affinity towards acidic sites, leading to the blockage of active sites [6]. Therefore, the use of a suitable catalyst is crucial for ensuring efficient operation at high temperatures.

1.1 $$2{\mathrm{CH}}_{3}\mathrm{OH}\to {\mathrm{CH}}_{3}{\mathrm{OCH}}_{3}+H_{2}O;\;\Delta H_{298K}=-22.6\mathrm{kJ}/\mathrm{mol}$$

The methanol conversion to DME and hydrocarbons used to be selectively carried out on the acid catalysts such as gamma alumina [7,8], zeolites [9,10] and silicoaluminophosphate [11,12]. Topology of the zeolites has been identified as the influencing parameter for product selectivity. Small pore size zeolites (2 to 10 Å) were found to be suitable for the selective products but associated with the slow diffusion constraint [13]. Aloise *et al*. reported the desilicated ZSM5 with Si/Al = 22.5 was more active than the parent sample [14]. The increase in pore diameter of the mesopores due to desilication was attributed to the enhanced activity and carbon deposition suppression. Moreover, the textural properties were more influential to the formation of selective products such as poly-methylbenzenes rather than acidity. Zhao *et al*. stated that the beta zeolite with much higher Si/Al ratio between 136 and 340 along with lower acid site density was suitable for selective methanol to propene (MTP) reaction [15]. Large pore size and high reaction temperature were summarized as the prime reasons for aromatic suppression and cracking of heavier olefins, respectively [15]. However, poor stability of the zeolite was an associated concern, with an observed 20% decrease in methanol conversion in 16 h. Thus the stability issue of the beta zeolite during methanol dehydration needs to be addressed.

The current study demonstrated the methanol dehydration reaction on beta zeolite sample. Beta zeolite was synthesized by hydrothermal crystallization and employed for the production of DME and light hydrocarbons. The effect of temperature on the structural stability of beta zeolite and product distribution was evaluated by a series of activity and characterization experiments. The reaction temperature is a crucial parameter for methanol dehydration and molecular sieve stability. Sabour *et al*. reported equilibrium limitations for the methanol dehydration to DME reaction between 250 and 400°C for Al-HMS catalysts [16]. However, the used catalysts were not examined thoroughly. Moreover, changes in the pore structure due to dealumination were also suggested by the DFT study, previously [17]. Pore structure and crystallinity change was supposed to have a significant influence on the product distribution. Changes in the catalytic properties and their correlation with the activity could give a proper justification for the temperature effects. Moreover, the stability investigation of the current beta zeolite sample at an elevated temperature region is targeted. The efficient use of beta zeolite material is reported with significant activity, stability and reusability.

## 2. Material and methods

### 2.1. Catalyst preparation

Beta zeolite molecular sieve was synthesized by a hydrothermal crystallization method using an organic template. The initial gel mixture comprised mole ratios of SiO_2_/Al_2_O_3_ = 28.5, H_2_O/SiO_2_ = 23.8, TEA^+^/SiO_2_ = 0.42 and Na_2_O/SiO_2_ = 0.091. The reagents used were sodium aluminate (43.8% Al_2_O_3_, 39.0% Na_2_O), silica sol (40% SiO_2_), tetraethylammonium hydroxide (TEAOH, 30% wt/wt aqueous solution), sodium hydroxide (NaOH, AR) and deionized water. A precursor solution of Si and Al was prepared by dissolving the desired amount of sodium aluminate and silica sol in aqueous sodium hydroxide solution. TEAOH was used as the structure directing agent and mixed with 40% v/v aqueous H_2_SO_4_ solution. Both the solutions were vigorously mixed to form a homogeneous mixture. The final homogeneous reaction mixture was transferred into stainless steel autoclave and then heated at 140°C. The extent of crystallization was monitored as a function of the crystallization period. The progress in crystallization was established from the degree of crystallinity, kinetics of crystallization, and different phases obtained at different crystallization periods. On the basis of the integrated area of the XRD peak appearing at 2*θ* = 22.4°, the XRD pattern of the sample obtained after a crystallization period of 72 h has exhibited the most crystalline and pure zeolite beta phase, free from any amorphous contribution. The final solid product was centrifuged and washed thoroughly with deionized water and then dried at 120°C in a static air oven for 12 h. In order to remove the organic template, an as-synthesized sample was calcined at 550°C for 10 h under the air environment. During the calcination, the air flow was kept at 20 ml min^−1^. The calcined sample was further subjected to repetitive ion-exchange using 1 M ammonium chloride solution (in the proportion of 15 ml per gm of solid) at 80°C for 6 h. Excess salt was washed by deionized water until there were no detectable chloride ions. The samples were then dried overnight at 120°C in static air followed by calcination at 550°C for 6 h under flowing air to convert them into protonic forms. The protonic form of the sample was shaped into extrudates by mixing 40 wt.% amorphous silica (dry basis) as a binder. Acetic acid (3%) was added to this mixture until it formed a homogeneous paste. It was further peptized until the required consistency was achieved. The homogeneous paste was then subjected to extrusion by using 1.2 mm die disc. Finally, the extrudates were dried and then calcined at 550°C for 5 h.

### 2.2. Characterization

The catalytic properties were investigated by several characterization techniques. Surface area and pore structure by N_2_ adsorption/desorption, structural properties by the XRD, and acidic properties by NH_3_-TPD experiments. The surface morphology was analysed by FESEM, and TEM experiments. The deposited carbonaceous species quantification was carried out by the TGA and CHNS experiment. Solid product verification was performed by ^1^H NMR spectroscopy.

### 2.3. Activity test

**Figure 1.**
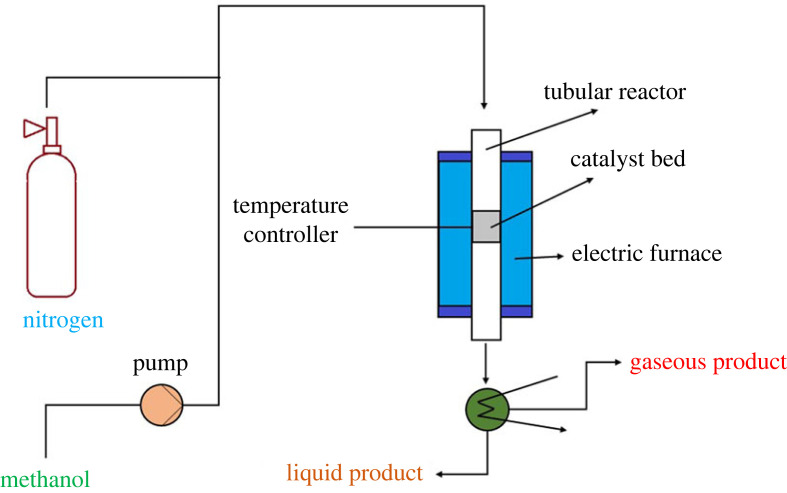
Schematic diagram of the experimental setup.

The performance evaluation of the prepared sample was conducted in a tubular quartz fixed bed reactor unit. A schematic diagram of the experimental setup is shown in figure 1. 8 g of the catalyst was loaded in the reactor with the help of quartz wool supported on the notch. The catalytic investigation was carried out between 280 and 450°C and a WHSV of 1.19 g_MeOH_ g_cat_^−1^ h^−1^. All reactions were carried out at atmospheric pressure, using nitrogen as the inert gas. The reaction products, gas and liquid, were analysed by refinery gas analyzer (RGA, Agilent Technologies 7890B) and gas chromatography (GC, Agilent Technologies 7890A), respectively. The samples were collected at different time intervals and analysed for methanol conversion and product distribution. The solid product was analysed by ^1^H NMR at the end of each reaction. The turnover frequency (TOF) was calculated by the following equation [18].

$$\mathrm{TOF}=\frac{\left({\mathrm{WHSV}}^{*}*x_{\mathrm{MeOH}}\right)}{\left({\mathrm{NH}}_{3}\;\mathrm{uptake}*X2\right)}$$

where, $WHSV^{*}$ and $x_{MeOH}$ are the molar hourly space velocity (µmol_MeOH_ g_cat_^−1^ h^−1^) and fractional conversion of methanol, respectively. Additionally, the $NH_{3}\;uptake$ data is used for the fresh beta zeolite extrudates. X2 is the fraction of strong acid sites reported in table 3.

## 3. Results and discussion

### 3.1. Fresh sample characterization

**Figure 2.**
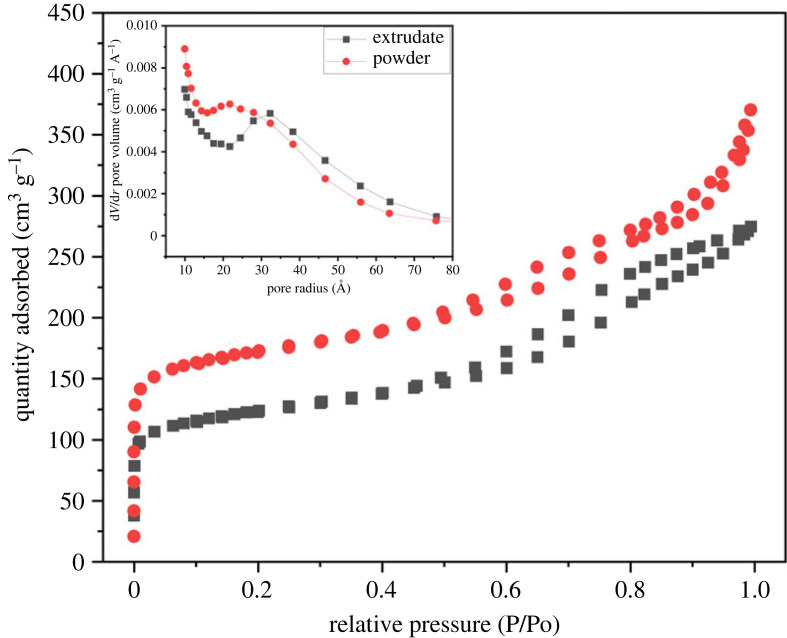
Isotherm and pore size distribution of the fresh β-zeolite.

Figure 2 shows N_2_ adsorption–desorption isotherm and pore size distribution of freshly calcined beta zeolite sample. Both the powdered and extrudate samples showed Type IV isotherm with a hysteresis loop that indicated the presence of mesoporous structure [19]. The total adsorbed N_2_ amount on the extrudates was lower than the powdered sample. The mixing of amorphous silica for shaping the powdered beta zeolite was the inherent reason behind the lower absorptivity [20]. Moreover, the inset plot shows the availability of both micro- and mesopores with the majority pore radius between 10 and 80 Å. The extrudate sample was taken as the basis with BET surface area and pore volume of 379.5 m^2^ g^−1^ and 0.42 cm^3^ g^−1^, respectively. Moreover, the micropore volume was evaluated as 0.11 cm^3^ g^−1^.

The X-ray diffraction pattern of the as-synthesized and calcined beta-zeolite powder and extrudates is reported in electronic supplementary material, figure S1. Crystalline structure was observed with distinct signals at 2*θ* = 7.8° and 22.4° [21]. The as-prepared parent sample matches well with the previously reported beta zeolite sample confirming the well-crystalline phase with no other impurity [18,19,22]. The XRD patterns of the protonic forms after post-synthesis treatment also showed identical profiles indicating no phase change or structural damage except some alteration in the relative peak intensities with no shift in the peak positions. The extent of crystallinity of the samples was calculated by the peak area at 22.4°and reported in table 1. No significant change in the crystallinity of the beta zeolite powder was observed by mixing the SiO_2_ binder. However, the crystallite size was slightly decreased from 55.9 nm to 52.8 nm while adding the SiO_2_ binder.

**Table 1.** Textural properties of fresh and used beta zeolite samples calcined at 550°C.

sample	reaction temperature (°C)	^a^surface area (m^2^/g)	^a^pore volume (cm^3^/g)	^a^micropore volume (cm^3^/g)	^b^crystallinity (%)	^d^crystallite size (nm)
fresh (powder)	—	544.5	0.54	0.17	^c^100.0	55.9
fresh (extrudate)	—	379.5	0.42	0.11	98.8	52.8
used (extrudate)	300	360.9	0.41	0.10	84.8	55.6
	350	319.8	0.38	0.09	87.9	52.8
	400	336.9	0.41	0.09	84.4	55.7
	450	371.6	0.42	0.10	29.4	57.2

^a^Obtained from N_2_ adsorption–desorption analysis.

^b^Based on XRD peak area.

^c^Assumed to have maximum crystallinity.

^d^Calculated by Scherrer equation.

**Figure 3.**
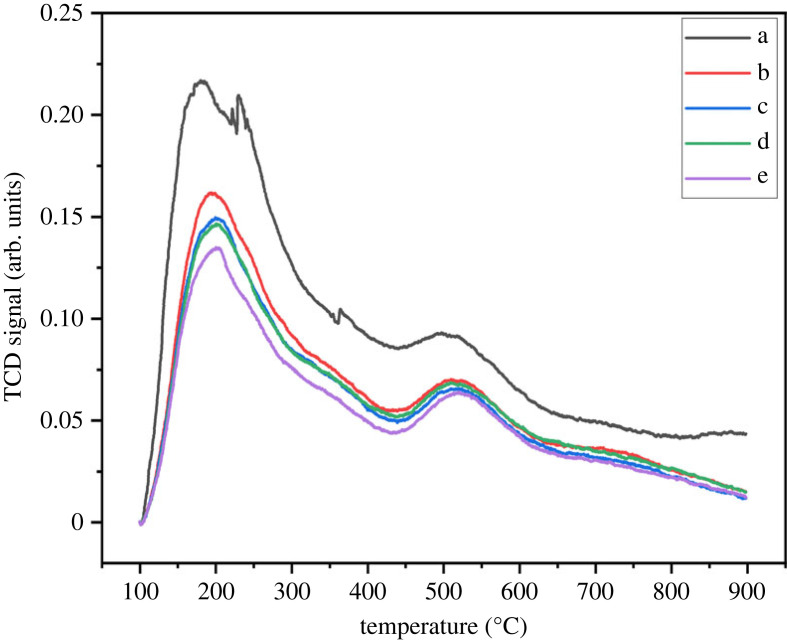
NH_3_-TPD profile (*a*) fresh and (*b*–*e*) used beta zeolite sample. Reaction conditions for used sample; pressure (1 bar), WHSV (1.19 g_MeOH_ g_cat_^−1^ h^−1^), temperature (*b*) 300°C (*c*) 350°C (*d*) 400°C (*e*) 450°C.

The acidic character of fresh beta zeolite was analysed by NH_3_ TPD experiment. Figure 3 shows two evident ammonia desorption peaks centred at 195°C and 510°C. The former was attributed to weak acid sites and the latter to strong acid sites [10,23]. Moreover, the major fraction of acid sites was weak acids, as reported in table 2.

**Table 2.** Comparison of the performance of prepared sample with the previous literature.

sample	temperature (°C)	pressure (bar)	WHSV (g_MeOH_ g_cat_^−1^ h^−1^)	MeOH conversion (%)	${\mathrm{TOF}}_{\mathrm{MeOH}}$ (h^−1^)	DME selectivity (%)	C2-C4 selectivity (%)	HMB selectivity (%)	ref.
beta zeolite	280	1	1.19	66.8	679.8	47.7	0.1	1.6	This work
	300			72.9	741.9	47.9	0.1	2.9	
	350			78.9	802.9	37.2	0.6	4.4	
	400			82.1	835.5	27.1	4.5	6.2	
	450			31.3	318.5	22.8	14.7	5.6	
SAPO-34	450	1	2.37	80.8	Not available	49.6	47.0	Not identified	[26]
beta zeolite	200	1	—	47	Not available	68	Not identified	Not identified	[21]
tungstosilicic acid (HSiW)	180	1	1.11	38	Not available	Not available	Not available	Not available	[34]
	200	1	1.11	50	Not available	Not available	Not available	Not available	
beta zeolite	240	1	4.5	48	185.0	98	—	—	[18]
bagasse fly ash derived beta zeolite	275	1	10.27	approximately 80	Not available	approximately 41	—	—	[25]
ZSM5	200	1	2	67	65	Not available	Not available	Not available	[14]
ZSM-5/Al_2_O_3_	200	1.2	2.6	46.8 ± 4.0	—	100	—	—	[27]
SBA-15	300	1	—	approximately 81	—	approximately 100	—	—	[28]

**Figure 4.**
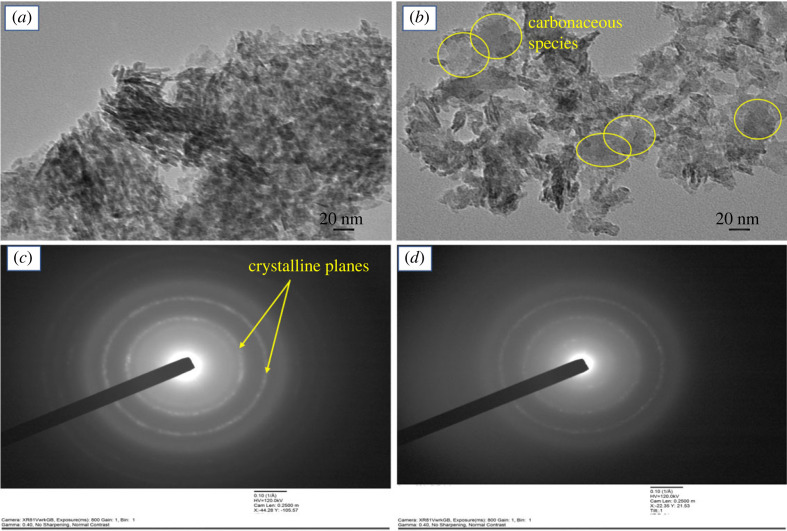
TEM image and SAED pattern of (*a*,*c*) fresh and (*b*,*d*) used beta zeolite sample. Reaction conditions for used sample; pressure (1 bar), WHSV (1.19 g_MeOH_ g_cat_^−1^ h^−1^), temperature (450°C).

The high-resolution surface morphology was investigated by TEM and reported in figure 4. The fresh beta zeolite shows regular and uniform distribution of the particles. Moreover, the SAED pattern suggested the polycrystalline structure of the fresh beta-zeolite sample.

### 3.2. Sample activity assessment

**Figure 5.**
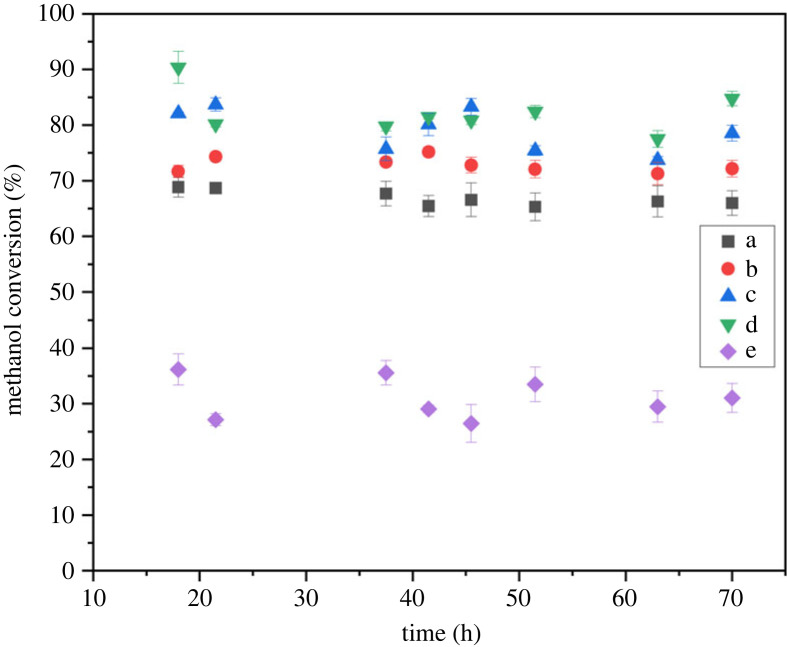
Methanol conversion data for the beta zeolite sample. Reaction conditions: pressure (1 bar), WHSV (1.19 g_MeOH_ g_cat_^−1^ h^−1^), temperature (*a*) 280°C (*b*) 300°C (*c*) 350°C (*d*) 400°C (*e*) 450°C.

Figure 5 shows the methanol conversion with time at various reaction temperatures. The time-on-stream (TOS) data shows almost stable conversions at all the investigated temperatures. An increase in methanol conversion was observed with the increase in reaction temperature from 280 to 400°C. The increase in the methanol conversion with temperature is consistent with the previously observed trend [18,24]. By contrast, a rapid decrease in the methanol conversion from 82.1% to 31.3% was observed while increasing the temperature from 400 to 450°C.

**Figure 6.**
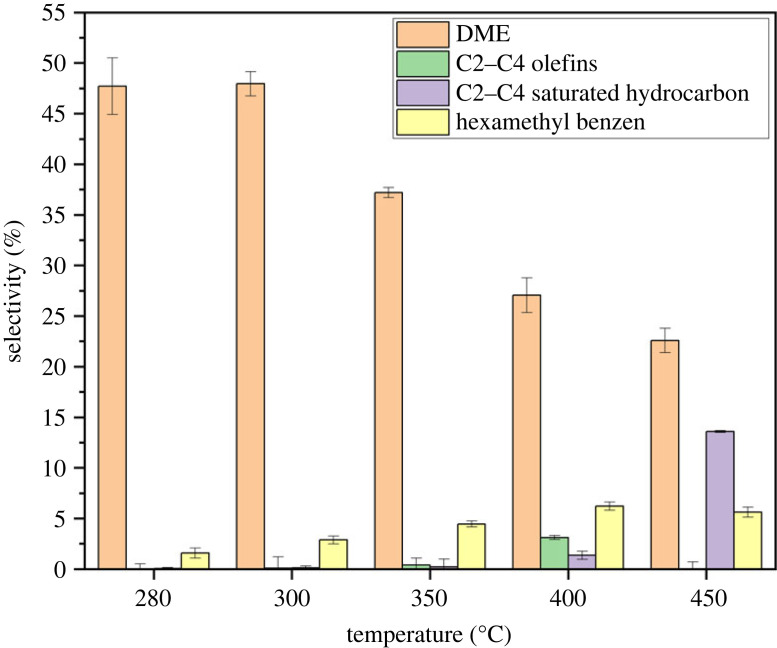
Product selectivity data for the beta zeolite sample averaged over the time-on-stream. Reaction conditions: pressure (1 bar), WHSV (1.19 g_MeOH_ g_cat_^−1^ h^−1^).

Liquid and gaseous products of the reactions were quantified using gas and liquid chromatography. Figure 6 shows the product selectivity averaged across the TOS studied. The results demonstrated that the synthesized beta zeolite catalyst is selective to DME between 280 and 300°C. Furthermore, a declining trend in DME selectivity was seen as temperature increased. Thus, the ideal temperature for methanol dehydration to DME was 300°C with maximal methanol conversion and dimethyl ether selectivity (%).

Saturated and unsaturated hydrocarbons were also obtained along with DME and with increasing selectivity with temperature. Figure S2 in the electronic supplementary material shows the product distribution of C1 to C4 hydrocarbons at each investigated temperature. Reaction temperature plays a pivotal role in hydrocarbon selectivity. C2 hydrocarbon formation was feasible at 300°C. However, C4 hydrocarbon percentage was maximized at 450°C, as shown in electronic supplementary material, figure S2d. Hexamethyl benzene (HMB) was also formed at all reaction temperatures with a maximum selectivity of 5.6% at 450°C. The ^1^H NMR spectrum in electronic supplementary material, figure S3 confirms the single component formation as the solid product. The product selectivity suggests the occurrence of the dual-cycle mechanism which propagates both the aromatic and olefin-based cycle [13].

The performance comparison of the present beta zeolite with the available literature is not straightforward because of the different reaction conditions. However, the comparison can be carried out with reasonable extrapolation. Table 2 contains the activity data of the previously reported molecular sieves for methanol dehydration reactions. The synthesized beta zeolite sample in the present study showed higher methanol conversion than the previously reported sample [18,21,25]. Although the reaction temperature was lower in the previous literature, the current beta zeolite sample might continue to have superior methanol conversion at a similar temperature. Previously investigated SAPO-34 by Álvaro-Muñoz *et al*. has higher methanol conversion than the current beta zeolite sample [26]. However, the catalyst showed a rapid decrease in methanol conversion after 5 h of activity test. The current beta zeolite sample showed higher methanol conversion than previously tested membrane-associated ZSM-5/Al_2_O_3_ [27]. Macina *et al*. reported around 81% methanol conversion with nearly 100% DME selectivity for mesoporous silica materials [28]. However, the study was limited in terms of addressing the stability of the catalyst. TOF_MeOH_ was also calculated to understand the activity per strong acid site. The present beta zeolite showed significantly high activity of acidic sites with TOF_MeOH_ of 679.8 h^−1^ at 280°C. The obtained TOF_MeOH_ is much higher than previously reported beta zeolite by Catizzone *et al*. [18]. Thus, the current zeolite sample consists of better activity toward methanol dehydration to DME with 70 h of stability.

### 3.3. Used sample characterization

#### 3.3.1. Surface morphology and coking analysis

**Figure 7.**
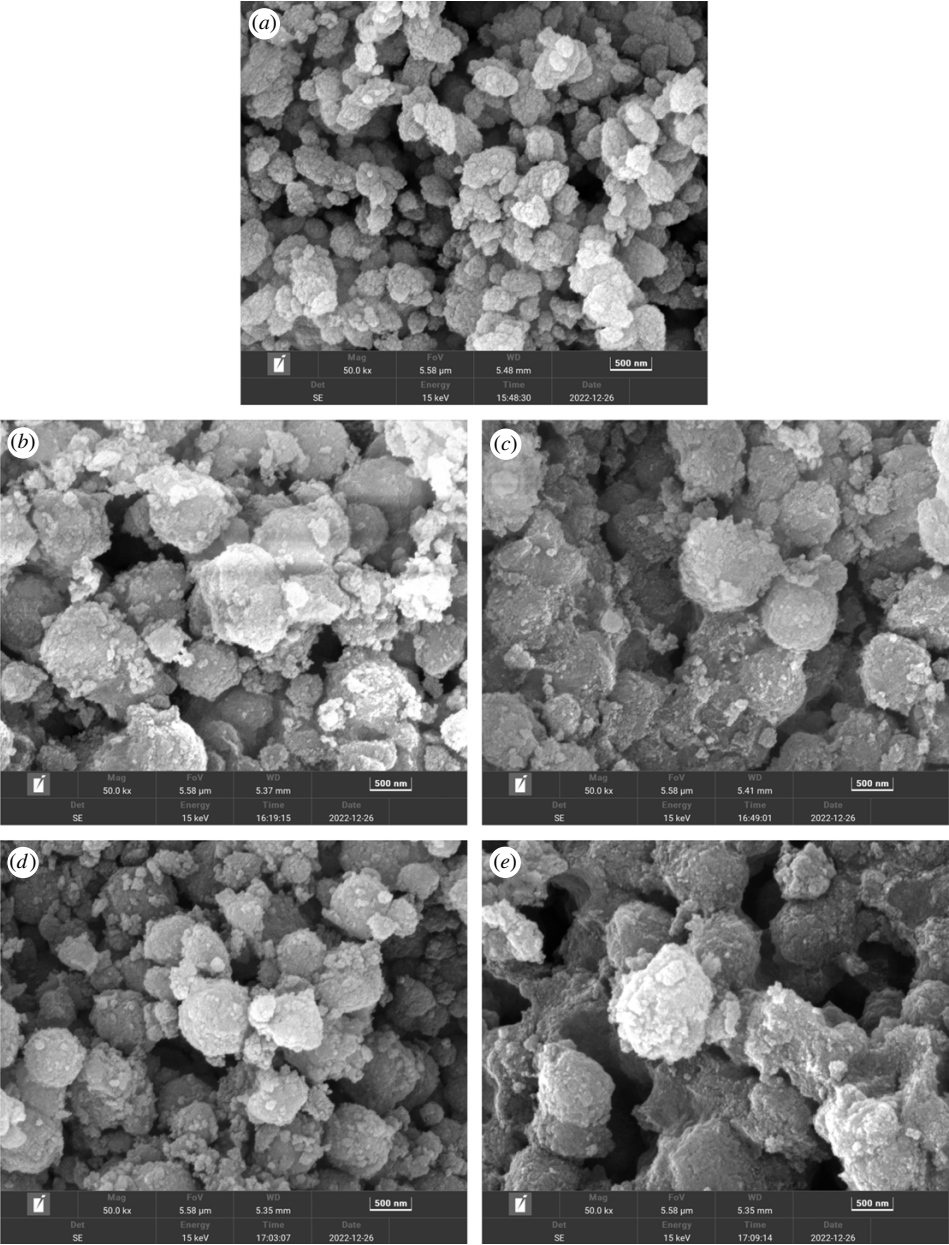
FESEM images of (*a*) fresh and (*b*–*e*) used beta zeolite sample. Reaction conditions for used sample; pressure (1 bar), WHSV (1.19 g_MeOH_ g_cat_^−1^ h^−1^), temperature (*b*) 300°C (*c*) 350°C (*d*) 400°C (*e*) 450°C.

Figure 7 shows the surface images of fresh and used beta zeolite samples. The roughness of the fresh sample was more as compared to the used samples. Additionally, bigger morphology appeared in used samples, which was possibly due to the amorphous SiO_2_ addition during extrudate synthesis. The elemental composition of selected areas of fresh beta zeolite is reported in electronic supplementary material, figure S4a. The Si/Al weight ratio was observed as 17.0, which matches well with the nominal ratio of 16.9. Furthermore, deposited carbon was confirmed in each used sample, although their appearance was not verified during imaging. The deposited carbon amount increased with an increase in the reaction temperature with a maximum amount of 38.3% at 450°C, as reported in table 3.

**Table 3.** Acidic properties and carbon deposition data of the fresh and used beta zeolite samples.

sample	reaction temperature (°C)	^a^total NH_3_ uptake (µmol g^−1^)	^a,b^T1 (°C)	^a,c^X1	^a,d^T2 (°C)	^a,e^X2	carbon (wt. %)		Wt. loss in TGA (%)
							CHNS	EDX	
fresh	-	522.0	172	0.93	520	0.07	0.0	0.0	4.7
used	300	477.5	191	0.90	524	0.10	22.8	28.0	13.3
	350	456.5	199	0.90	524	0.10	25.6	31.0	17.2
	400	449.4	200	0.89	521	0.11	27.2	37.1	24.1
	450	442.2	200	0.89	521	0.11	28.0	38.3	25.3

^a^Obtained from NH_3_ TPD.

^b^Temperature of weak acid peak.

^c^Fraction of weak acid site.

^d^Temperature of strong acid peak.

^e^Fraction of strong acid site.

**Figure 8.**
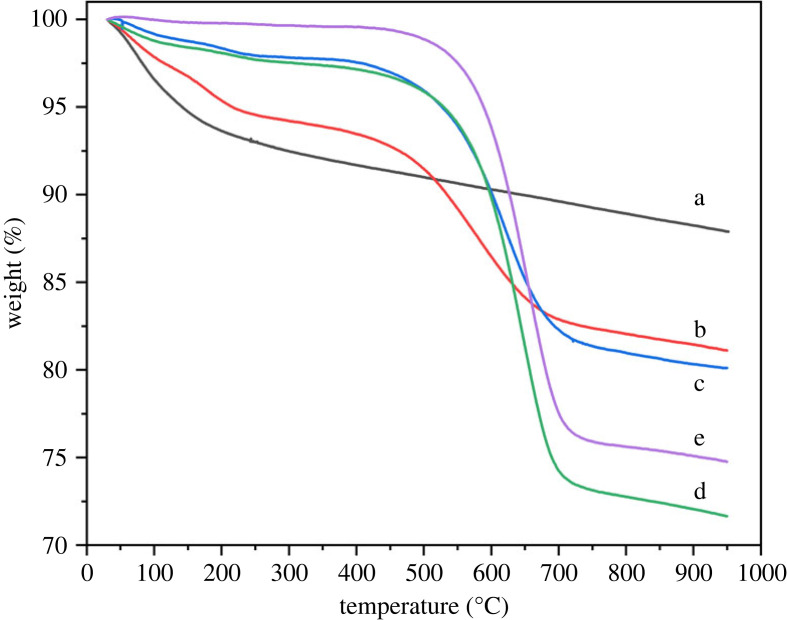
TG profile of (*a*) fresh and (*b*–*e*) used beta zeolite sample. Reaction conditions for used sample; pressure (1 bar), WHSV (1.19 g_MeOH_ g_cat_^−1^ h^−1^), temperature (*b*) 300°C (*c*) 350°C (*d*) 400°C (e) 450°C.

The deposited carbon on used samples was estimated using thermogravimetry analysis (TGA). The TG profile in figure 8 shows a decrease in sample weight with an increase in temperature. Thermogravimetry data of the fresh sample was also included for comparison. The weight loss between room temperature to 200°C is due to removal of moisture adsorbed in the sample [29]. The weight loss between 200 and 800°C is attributed to burning of coke which was deposited during the reaction. All the used samples showed higher weight loss as compared to the fresh sample. Moreover, deposited carbon amount increased with an increase in the reaction temperature.

Table 3 shows the deposited carbon amount in the used samples by the CHNS experiment. An increase in the carbon amount was confirmed with an increase in reaction temperature. Although the absolute values are different, the trend of increase in carbon deposition with temperature is in agreement with the TGA and EDX data. Catizzone *et al*. stated the carbon deposition in the form of poly-methylbenzenes during methanol dehydration [18]. In fact, the current study showed single hexamethyl benzene (HMB) formation as a solid product. Although the HMB deposition was attributed to the prime reason for catalyst deactivation, the present beat zeolite appeared to have a smaller pore structure to avoid holding HMB.

The carbon deposition during methanol dehydration was considered as a significant reason for the activity loss. In fact, a previous study by Catizzone *et al*. reported a decrease in methanol conversion from 84% to 48% in 60 h of TOS on a beta zeolite sample and referred to coking [18]. Nevertheless, the current study reveals that the beta zeolite exhibits nearly consistent activity for up to 70 h of time on stream (TOS). Furthermore, it has been observed that a significant decline in crystallinity is the primary factor contributing to the decrease in activity at higher reaction temperatures.

#### 3.3.2. Regeneration of used samples

Used samples after 70 h of the activity test were investigated to identify the occurred changes in textural and physico-chemical properties. Prior to the investigations, used samples were calcined in the still-air environment at 550°C for 5 h. After that, the sample was cooled down to room temperature and used for characterization investigations.

The N_2_ adsorption–desorption isotherm of used samples after regeneration in electronic supplementary material, figure S5 shows almost identical type IV isotherm as of the fresh beta zeolite extrudate. Furthermore, table 1 shows a decrease in surface area with an increase in the reaction temperature up to 350°C when compared to the fresh sample. Further increase in reaction temperature leads to an increase in the surface area and pore volume. The pore structure appeared to change at elevated temperatures. For added clarity, pore size distribution of the used samples is reported in electronic supplementary material, figure S6. All the regenerated samples sustained the microporous-mesoporous structure. However, used samples after activity tests at 400 and 450°C showed a shift in the pore size distribution to the widened size. Dealumination of the used beta zeolite due to steaming at elevated temperature appeared to be related to pore broadening and increment in the pore volume. Steam treatment of zeolites has been reported as the prominent method for dealumination of small pore zeolites [17,30,31]. Raoof *et al*. also reported a rapid decrease in the methanol conversion with TOS for γ-Al_2_O_3_ with the 20% H_2_O co-feed [8]. The observed increase in carbon deposition at elevated temperatures (as reported in table 3) could be related to the pore-broadening phenomena. The widened pore pockets hold the HMB with a subsequent increase in the deposited quantity [18]. Thus, the pore broadening appeared to happen at higher reaction temperatures along with the increase in carbonaceous species deposition. Nonetheless, the microporosity of all the regenerated samples remains the same.

**Figure 9.**
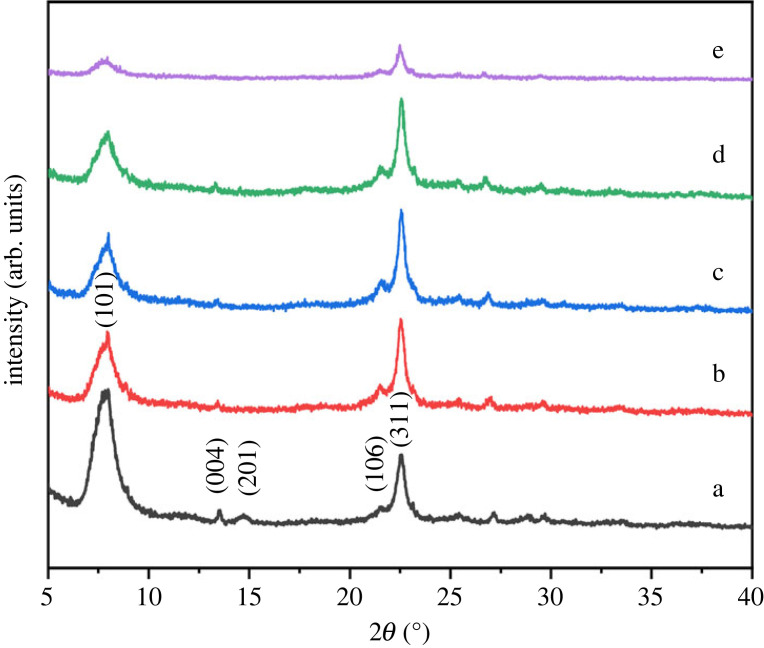
XRD pattern of (*a*) fresh and (*b*–*e*) used beta zeolite sample. Reaction conditions for used sample; pressure (1 bar), WHSV (1.19 g_MeOH_ g_cat_^−1^ h^−1^), temperature (*b*) 300°C (*c*) 350°C (*d*) 400°C (*e*) 450°C.

The phase purity and crystallinity of used samples were determined by powder X-ray diffraction. The diffractogram of used catalysts in figure 9 showed diffraction peaks at the same position as those observed for the fresh sample. The crystallinity of used samples was calculated by comparing the integrated peak area at 2*θ* = 22.4° to that of the parent beta zeolite. Freshly calcined beta zeolite powder was assumed to have the most crystalline structure with 100% crystallinity. Compared to the fresh catalyst, used catalysts showed a decrease in crystallinity, with minor exceptions, as reported in table 1. Moreover, a gradual decrease in diffraction peak area at 2*θ* = 7.8° is evident and affirms the decreased crystallinity with increase in reaction temperature. Furthermore, the crystalline structure appeared excessively hampered at elevated temperatures. The used beta zeolite after activity test at 450°C showed a decrease in crystallinity of 70.6%. The loss in crystallinity affirms the hypothesis of dealumination during methanol dehydration [30]. Lower methanol conversion at 450°C also seemed to be related to the loss in crystallinity. Moreover, all the used catalysts showed similar crystal sizes as compared to fresh beta zeolite.

The TEM image of used beta zeolite after activity test at 450°C showed significant deviation from the fresh sample, as shown in figure 4. Amorphous carbon deposition was observed on the catalyst surface and showed agreement with TGA and CHNS data [32]. Morphology of the used sample was also hampered and could be related to loss in crystallinity, as discussed above. The SAED pattern of used beta zeolite in figure 4*d* showed an evident loss in the polycrystalline structure. Thus, the activity drop at 450°C was due to carbon deposition. However, the loss in crystallinity can't be overlooked as the reason for activity loss.

Table 3 shows the total acidity of used samples tested at different temperatures. A decrease in total acidity was observed with an increase in reaction temperature. However, the fraction of strong acidic sites increased with temperature. The increase in C2-C4 selectivity is consistent with increase in the fraction of strong acid sites [18]. However, the role of weak acid sites in methanol dehydration and product selectivity cannot be avoided [25]. The acid sites, weak and strong, are important in the DME synthesis and should be maintained throughout the reaction. Niwa *et al*. stated that the weak acid site evaluation based on low temperature NH_3_ desorption peak would not be appropriate because of the involvement of physisorbed ammonia [33]. However, decreasing overall NH_3_ adsorption amount for the used samples is consistent with a decrease in the DME selectivity. Thus, at elevated reaction temperatures, the structural properties of beta zeolite were mildly hampered and could be regenerable successfully after calcination.

#### 3.3.3. Regeneration cycle test

**Figure 10.**
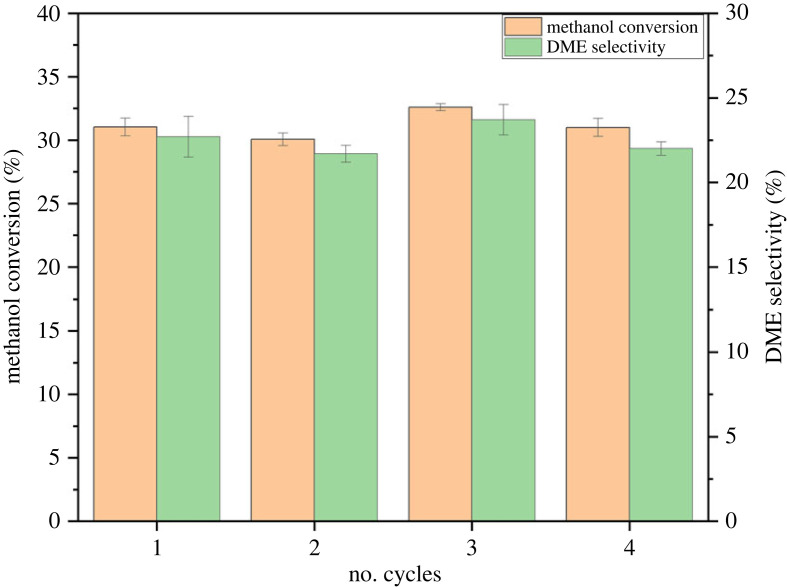
Methanol conversion and DME selectivity data for regeneration cycle test. Regeneration condition; calcination (550°C); reaction conditions for regenerated sample; pressure (1 bar), WHSV (1.19 g_MeOH_ g_cat_^−1^ h^−1^), temperature (450°C).

The used beta zeolite after 70 h intervals of activity test at 450°C was further exposed to regeneration test since the propensity of deactivation was more due to significant carbon deposition, as reported above. After each 70 h cycle, the used sample was regenerated by calcining in still air at 550°C for 5 h. The regenerated sample was again tested for methanol dehydration reaction for longer TOS. Figure 10 shows the methanol conversion and DME selectivity data for four reaction cycles. Thus, the current beta zeolite showed stable activity up to a consolidated TOS of 280 h.

#### 3.3.4. Reaction rate expression

In order to describe the methanol dehydration process, power-law kinetics was used for the beta zeolite catalyst with estimated parameters not only for the consumption of methanol but also for generation of H_2_O and DME. The experimental data for kinetic study were obtained for the methanol dehydration reaction at different temperatures (280^⚬^C, 300^⚬^C, 325^⚬^C, 350^⚬^C, 375^⚬^C, and 400^⚬^C) and a WHSV variation between 1.49 and 0.99 g_MeOH_ g_cat_^−1^ h^−1^. The experimental data was fitted in the power-law model equation in the nonlinear regression method by using Polymath software. The estimated rate constant and other parameters are given in table 4. During the integral method of analysis the graph was plotted to satisfy the Arrhenius equation. Electronic supplementary material, figure S7 suggested the activation energy of 4.2 kJ mole^−1^ with frequency factor 7.73 g_MeOH_/g_cat_.h.(atm)^0.52^.

**Table 4.** Summary of the kinetic model.

S.N.	kinetic equation	rate constant (k)	a	b	c	*R*^2^
1	$-r=kp_{\mathrm{MeOH}}^{a}p_{H2O}^{b}p_{\mathrm{DME}}^{c}$	3.2	0.78	−1.7	1.44	0.99

## 4. Conclusion

The synthesized beta zeolite by hydrothermal crystallization method results in molecular sieve with significant surface area, micro-mesoporosity and crystallinity. Methanol dehydration to DME was efficiently carried out with sustained stability. The increase in reaction temperature showed an increase in methanol conversion and a decrease in DME selectivity. Hydrocarbons between C1 to C4 range and hexamethyl benzene (HMB) were also identified as the reaction product. The molecular sieve crystallinity and porous structure stability were found to be pivotal for catalyst performance. The lower molecular weight C2 hydrocarbons were more selective at lower reaction temperatures. In contrast, C4 enrichment occurred at elevated temperatures. HMB was found as the single component as the solid product, along with the poly-methylbenzene deposition on the zeolite surface. At higher reaction temperatures, pore-broadening phenomena were observed, which increased the accumulation capacity of HMB. At elevated temperatures, apprehensions of structure deterioration arise due to steaming. Regenerability and recyclability with minimal loss in the structural properties promote the candidacy of the porous beta zeolite sample for methanol dehydration reaction.

## Ethical

This article does not report any study carried out on humans, human tissues or animals.

## Data Availability

The article's supporting data and digital research materials can be accessed at the following links. Dryad doi: https://doi.org/10.5061/dryad.q573n5tnx [35]. The datasets supporting this article have been uploaded as part of the electronic supplementary material [36].

## References

[1] 2023 Climate Change Conference in Glasgow (COP26). See https://www.un.org/en/climatechange/cop26 (accessed on 5 June 2023).

[2] Semelsberger TA, Borup RL, Greene HL. 2006 Dimethyl ether (DME) as an alternative fuel. J. Power Sources **156**, 497-511. (10.1016/j.jpowsour.2005.05.082)

[3] Sorenson SC. 2001 Dimethyl ether in diesel engines: progress and perspectives. J. Eng. Gas Turbine Power **123**, 652-658. (10.1115/1.1370373)

[4] Laugel G, Nitsch X, Ocampo F, Louis B. 2011 Methanol dehydration into dimethylether over ZSM-5 type zeolites: raise in the operational temperature range. Appl. Catal. A Gen. **402**, 139-145. (10.1016/j.apcata.2011.05.039)

[5] Zeng L, Wang Y, Mou J, Liu F, Yang C, Zhao T, Wang X, Cao J. 2020 Promoted catalytic behavior over γ-Al2O3 composited with ZSM-5 for crude methanol conversion to dimethyl ether. Int. J. Hydrogen Energy **45**, 16 500-16 508. (10.1016/j.ijhydene.2020.04.115)

[6] Semelsberger TA, Ott KC, Borup RL, Greene HL. 2005 Role of acidity on the hydrolysis of dimethyl ether (DME) to methanol. Appl. Catal., B **61**, 281-287. (10.1016/j.apcatb.2005.05.014)

[7] Alamolhoda S, Kazemeini M, Zaherian A, Zakerinasab MR. 2012 Reaction kinetics determination and neural networks modeling of methanol dehydration over nano γ-Al 2O 3 catalyst. J. Ind. Eng. Chem. **18**, 2059-2068. (10.1016/j.jiec.2012.05.027)

[8] Raoof F, Taghizadeh M, Eliassi A, Yaripour F. 2008 Effects of temperature and feed composition on catalytic dehydration of methanol to dimethyl ether over γ-alumina. Fuel **87**, 2967-2971. (10.1016/j.fuel.2008.03.025)

[9] Alharbi W, Kozhevnikova EF, Kozhevnikov Iv. 2015 Dehydration of methanol to dimethyl ether over heteropoly acid catalysts: the relationship between reaction rate and catalyst acid strength. ACS Catal. **5**, 7186-7193. (10.1021/acscatal.5b01911)

[10] Ferrarelli G, Giordano G, Migliori M. 2022 ZSM-5@Sil-1 core shell: effect of synthesis method over textural and catalytic properties. Catal. Today **390–391**, 176-184. (10.1016/j.cattod.2021.11.036)

[11] Minova IB, Barrow NS, Sauerwein AC, Naden AB, Cordes DB, Slawin AMZ, Schuyten SJ, Wright PA. 2021 Silicon redistribution, acid site loss and the formation of a core–shell texture upon steaming SAPO-34 and their impact on catalytic performance in the Methanol-to-Olefins (MTO) reaction. J. Catal. **395**, 425-444. (10.1016/j.jcat.2021.01.012)

[12] Das J, Bhattacharya A, Goswami TK, Roy SK. 1991 Conversion of methanol on silicoaluminophosphate-formation of hexamethylbenzene and olefins. J. Chcm. Tech. Biotechnol. **50**, 13-16. (10.1002/jctb.280500103)

[13] Liu Z, Dong X, Zhu Y, Emwas AH, Zhang D, Tian Q, Han Y. 2015 Investigating the Influence of Mesoporosity in Zeolite Beta on Its Catalytic Performance for the Conversion of Methanol to Hydrocarbons. ACS Catal. **5**, 5837-5845. (10.1021/acscatal.5b01350)

[14] Aloise A et al. 2020 Desilicated ZSM-5 zeolite: Catalytic performances assessment in methanol to DME dehydration. Microporous Mesoporous Mater. **302**, 110198. (10.1016/j.micromeso.2020.110198)

[15] Zhao X, Wang L, Li J, Xu S, Zhang W, Wei Y, Guo X, Tian P, Liu Z. 2017 Investigation of methanol conversion over high-Si beta zeolites and the reaction mechanism of their high propene selectivity. Catal. Sci. Technol. **7**, 5882-5892. (10.1039/c7cy01804e)

[16] Sabour B, Peyrovi MH, Hamoule T, Rashidzadeh M. 2014 Catalytic dehydration of methanol to dimethyl ether (DME) over Al-HMS catalysts. J. Ind. Eng. Chem. **20**, 222-227. (10.1016/j.jiec.2013.03.044)

[17] Malola S, Svelle S, Bleken FL, Swang O. 2012 Detailed reaction paths for zeolite dealumination and desilication from density functional calculations. Angew. Chem. - Int. Ed. **51**, 652-655. (10.1002/anie.201104462) 22147388

[18] Catizzone E, Aloise A, Migliori M, Giordano G. 2017 From 1-D to 3-D zeolite structures: performance assessment in catalysis of vapour-phase methanol dehydration to DME. Microporous Mesoporous Mater. **243**, 102-111. (10.1016/j.micromeso.2017.02.022)

[19] Li J, Liu H, An T, Yue Y, Bao X. 2017 Carboxylic acids to butyl esters over dealuminated-realuminated beta zeolites for removing organic acids from bio-oils. RSC Adv. **7**, 33 714-33 725. (10.1039/c7ra05298g)

[20] Trypolskyi A, Zhokh A, Gritsenko V, Chen M, Tang J, Strizhak P. 2021 A kinetic study on the methanol conversion to dimethyl ether over H-ZSM-5 zeolite. Chemical Papers **75**, 3429-3442. (10.1007/s11696-021-01586-y)

[21] Catizzone E, Giglio E, Migliori M, Cozzucoli PC, Giordano G. 2020 The effect of zeolite features on the dehydration reaction of methanol to dimethyl ether: Catalytic behaviour and kinetics. Materials **13**, 1-15. (10.3390/ma13235577) PMC7730933 33297548

[22] Catizzone E, Aloise A, Migliori M, Giordano G. 2015 Dimethyl ether synthesis via methanol dehydration: effect of zeolite structure. Appl. Catal. A Gen. **502**, 215-220. (10.1016/j.apcata.2015.06.017)

[23] Palomo J, Rodríguez-Cano MA, Berruezo-García J, Rodríguez-Mirasol J, Cordero T. 2022 Efficient methanol dehydration to DME and light hydrocarbons by submicrometric ZrO2-ZSM-5 fibrillar catalysts with a shell-like structure. Fuel **315**, 123283. (10.1016/j.fuel.2022.123283)

[24] Catizzone E, Aloise A, Giglio E, Ferrarelli G, Bianco M, Migliori M, Giordano G. 2021 MFI vs. FER zeolite during methanol dehydration to dimethyl ether: The crystal size plays a key role. Catal. Commun. **149**, 106214. (10.1016/j.catcom.2020.106214)

[25] Assawasangrat P, Neramittagapong S, Pranee W, Praserthdam P. 2016 Methanol conversion to dimethyl ether over beta zeolites derived from bagasse fly ash. Energy Sources A: Recovery Util. Environ. Eff. **38**, 3081-3088. (10.1080/15567036.2015.1124945)

[26] Álvaro-Muñoz T, Márquez-Álvarez C, Sastre E. 2012 Use of different templates on SAPO-34 synthesis: Effect on the acidity and catalytic activity in the MTO reaction. Catal. Today **179**, 27-34. (10.1016/j.cattod.2011.07.038)

[27] Brunetti A, Migliori M, Cozza D, Catizzone E, Giordano G, Barbieri G. 2020 Methanol conversion to dimethyl ether in catalytic zeolite membrane reactors. ACS Sustain. Chem. Eng. **8**, 10 471-10 479. (10.1021/acssuschemeng.0c02557)

[28] Macina D, Piwowarska Z, Tarach K, Góra-Marek K, Ryczkowski J, Chmielarz L. 2016 Mesoporous silica materials modified with alumina polycations as catalysts for the synthesis of dimethyl ether from methanol. Mater. Res. Bull. **74**, 425-435. (10.1016/j.materresbull.2015.11.018)

[29] Rahman M, Infantes-Molina A, Hoffman AS, Bare SR, Emerson KL, Khatib SJ. 2020 Effect of Si/Al ratio of ZSM-5 support on structure and activity of Mo species in methane dehydroaromatization. Fuel **278**, 118290. (10.1016/j.fuel.2020.118290)

[30] Yoshioka T et al. 2022 Dealumination of small-pore zeolites through pore-opening migration process with the aid of pore-filler stabilization. Sci. Adv. **8**, eabo3093. (10.1126/sciadv.abo3093) 35731864 PMC9216521

[31] Valdiviés-Cruz K, Lam A, Zicovich-Wilson CM. 2017 Full Mechanism of Zeolite Dealumination in Aqueous Strong Acid Medium: Ab Initio Periodic Study on H-Clinoptilolite. J. Phys. Chem. C **121**, 2652-2660. (10.1021/acs.jpcc.6b09794)

[32] Hintsho N, Shaikjee A, Tripathi PK, Franklyn P, Durbach S. 2015 The effect of CO2 on the CVD synthesis of carbon nanomaterials using fly ash as a catalyst. RSC Adv. **5**, 53 776-53 781. (10.1039/c5ra06892d)

[33] Niwa M, Katada N. 2013 New method for the temperature- programmed desorption (TPD) of ammonia experiment for characterization of zeolite acidity: a review. Chem. Rec. **13**, 432-455. (10.1002/tcr.201300009) 23868494

[34] Peinado C, Liuzzi D, Ladera-Gallardo RM, Retuerto M, Ojeda M, Peña MA, Rojas S. 2020 Effects of support and reaction pressure for the synthesis of dimethyl ether over heteropolyacid catalysts. Sci. Rep. **10**, 8551. (10.1038/s41598-020-65296-3) 32444653 PMC7244519

[35] Chaudhary PK, Arundhathi R, Kasture MW, Samanta C, Vankayala R, Thota C. 2023 Data from: Temperature-dependent synthesis of dimethyl ether (DME) from methanol over beta zeolite: A novel approach to a sustainable fuel. Dryad Digital Repository. (10.5061/dryad.q573n5tnx) PMC10445025 37621656

[36] Chaudhary PK, Arundhathi R, Kasture MW, Samanta C, Vankayala R, Thota C. 2023 Temperature-dependent synthesis of dimethyl ether (DME) from methanol over beta zeolite: a novel approach to a sustainable fuel. Figshare. (10.6084/m9.figshare.c.6764118) PMC10445025 37621656

